# Elucidating the impact of boron fertilization on soil physico-chemical and biological entities under cauliflower-cowpea-okra cropping system in an Eastern Himalayan acidic Inceptisol

**DOI:** 10.3389/fmicb.2022.996220

**Published:** 2022-11-07

**Authors:** Ingudam Bhupenchandra, Anjali Basumatary, Anil K. Choudhary, Adarsh Kumar, Dibyendu Sarkar, Sunil Kumar Chongtham, Athokpam Herojit Singh, Elangbam Lamalakshmi Devi, S. S. Bora, Menaka Devi Salam, Manas Ranjan Sahoo, Bharat A. Gudade, Amit Kumar, Soibam Helena Devi, Bhabesh Gogoi, M. N. Harish, Gaurendra Gupta, Leitam Chanu Olivia, Yumnam Prabhabati Devi, Konsam Sarika, Shobit Thapa, Mahendra Vikram Singh Rajawat

**Affiliations:** ^1^ICAR-KVK Tamenglong, Indian Council of Agricultural Research–Research Complex for North–Eastern Hill Region, Manipur Centre, Imphal, Manipur, India; ^2^Department of Soil Science, Assam Agricultural University, Jorhat, Assam, India; ^3^Division of Agronomy, Indian Council of Agricultural Research–Indian Agricultural Research Institute, New Delhi, India; ^4^Division of Crop Production, Indian Council of Agricultural Research–Central Potato Research Institute, Shimla, India; ^5^ICAR-National Bureau of Agriculturally Important Microorganism, Mau, India; ^6^Department of Agricultural Chemistry and Soil Science, Bidhan Chandra Krishi Viswavidyalaya, Mohanpur, West Bengal, India; ^7^Multi Technology Testing Centre and Vocational Training Centre, College of Agricultural Engineering and Post Harvest Technology, Central Agricultural University, Ranipool, Sikkim, India; ^8^College of Agriculture, Central Agricultural University, Imphal, Manipur, India; ^9^ICAR RC for NEH Region, Sikkim Centre, Tadong, Sikkim, India; ^10^Regional Research Station, Indian Cardamom Research Institute, Spices Board, Tadong, Gangtok, India; ^11^Amity Institute of Microbial Technology, Amity University Uttar Pradesh, Noida, India; ^12^Central Horticultural Experiment Station, Indian Council of Agricultural Research–Indian Institute of Horticultural Research, Bhubaneswar, Odisha, India; ^13^Department of Crop Physiology, Assam Agricultural University, Jorhat, Assam, India; ^14^Farm Science Centre, Indian Council of Agricultural Research–Indian Institute of Horticultural Research, Kodagu, Karnataka, India; ^15^ICAR-Indian Grassland and Fodder Research Institute, Jhansi, Uttar Pradesh, India; ^16^Department of Agronomy, College of Agriculture, Central Agricultural University, Imphal, India; ^17^ICAR-KVK Chandel, ICAR RC for NEH Region, Manipur Centre, Imphal, Manipur, India; ^18^ICAR RC for NEH Region, Manipur Centre, Imphal, Manipur, India

**Keywords:** boron, Inceptisol, microbiological pools, microbial populations, microbial biomass carbon, soil enzymatic activities

## Abstract

Information on the role of boron (B) on soil physico-chemical and biological entities is scarce, and the precise mechanism in soil is still obscure. Present field investigation aimed to assessing the implication of direct and residual effect of graded levels of applied-B on soil biological entities and its concomitant impact on crop productivity. The treatments comprised of five graded levels of B with four replications. To assess the direct effect of B-fertilization, cauliflower was grown as a test crop wherein, B-fertilization was done every year. For assessment of succeeding residual effects of B-fertilization, cowpea and okra were grown as test crops and, B-fertilization was phased out in both crops. The 100% recommended dose of NPK (RDF) along with FYM was uniformly applied to all crops under CCOCS. Results indicated that the direct effect of B had the edge over residual effect of B in affecting soil physico-chemical and biological entities under CCOCS. Amongst the graded levels of B, application of the highest B level (2 kg ha^–1^) was most prominent in augmenting microbiological pools in soil at different crop growth stages. The order of B treatments in respect of MBC, MBN, and soil respiration at different crop growth stages was 2.0 kg B ha^–1^ > 1.5 kg B ha^–1^ > 1.0 kg B ha^–1^ > 0.5 kg B ha^–1^ > 0 kg B ha^–1^, respectively. Moreover, maximum recoveries of potentially mineralizable-C (PMC) and potentially mineralizable-N (PMN) were noticed under 2 kg B ha^–1^. Analogous trend was recorded in soil microbial populations at different crop growth stages. Similarly, escalating B levels up to 2 kg B ha^–1^ exhibited significantly greater soil enzymatic activities *viz.*, arylsulphatase (AS), dehydrogenase (DH), fluorescein diacetate (FDA) and phosphomonoesterase (PMA), except urease enzyme (UE) which showed an antagonistic effect of applied-B in soil. Greater geometric mean enzyme activity (GMEA) and soil functional diversity index were recorded under 2 kg B ha^–1^ in CCOCS, at all crop growth stages over control. The inclusive results indicated that different soil physico-chemical and biological properties CCOCS can be invariably improved by the application of graded levels of B up to 2 kg B ha^–1^ in an acid Inceptisol.

## Introduction

Boron (B) is a vital micronutrient that is indispensable for proper crop growth (Bhupenchandra et al., 2021a). Boron is a necessary micro-element for plant cell wall structural integrity and is involved in various plant processes like cell division, calcium utilization, pollen production, and anther development during the reproductive phase (Nadeem et al., 2019). Currently, the B deficits in soils are widespread globally causing B micronutrient malformations that impinge on agricultural production (Shorrocks, 1997; Liu, 2000; Choudhary et al., 2014; Behera et al., 2022). Due to excess deficiency symptoms manifested as an implication of B deficit, the assessment of key functions of B in plants has long been the main concern from a nutritional point of view. For plants, managing B is difficult because the optimum B range is narrow which can fluctuate from soil to soil (Gupta, 1993; Marschner, 1995). Normally, B averaged nearly 30 mg kg^–1^ soil depending on the main rock wherein its content varies extensively. Satisfactory B content for flora in soils is more or less 25 mg kg^–1^ (GreenFacts, 2002). Soil microbial biomass holds a vital role in nutrient-cycling, plant-pathogen suppression, the disintegration of debris, and decay of pollutants establishing the vibrant living entity of soil, and thus, attributing to ecological sustainability owing to their diverse existence, enormous effective genetic pools, catabolic adaptability, and stress tolerance ability in a holistic manner (Deluca et al., 2019). The dimension and activity of the microbial biomass determine the nutrient availability and production potential of the agro-ecosystems (Friedel et al., 1996; Singh et al., 2020, 2021). Therefore, it becomes obligatory to determine microbial biomass nitrogen (MBN). Since it becomes vital for the quantification of N-dynamics in agro-ecosystems as it controls the soil inorganic-N accessibility and loss and its contribution to the primary N-sources of potentially mineralizable-N (PMN) in the soil (Bonde et al., 1988). Microbial biomass carbon (MBC), MBN, and microbial respiration, have further garnered added interest owing to their sensitivity to crop management practices than the bulk soil organic matter (Awale et al., 2017). Soil microbes, the existing fraction of soil organic matter (SOM) acts as a transitory nutrient-sink and are accountable for unleashing nutrients from SOM for exploitation by plants (Bollag et al., 2017). Basal respiration (BR) and C-mineralization are ample indicators of microbial activity, depending on the substrate accessibility and the soil edaphic environment (Balota et al., 2003). On the whole, the CO_2_ respired during a year in terrestrial ecosystems is the consequence of C-mineralization of the minute active fraction pools, which are mainly accountable for unleashing nutrients in the soil (Brdar-Jokanović, 2020).

It is implicit to address that B acts a crucial function in the biological activities of living organisms as proven earlier by establishing the necessity of B for diatoms and cyanobacteria (Bonilla et al., 1997). Soil microorganisms are by far the most important producers of soil enzymes that perform many ecological processes such as bio-geochemical cycling and decomposing pollutants and debris from flora and fauna and the microbes (Goswami et al., 2017; Furtak and Gajda, 2018). Soil microbes are accountable for the transformation of SOM and soil nutrients (Mooshammer et al., 2014; Singh et al., 2020, 2021). The microbes and their enzymatic outputs are indispensable to plants, while plant roots generate organic substances that are vital to the populace expansion of microbes (Jjemba and Alexander, 1999). Soil microbial indices is touted as a substitute for organic carbon cycling and its related nutrients *viz.*, N, P, and S, signifying that elevated microbial action implied increased soil productivity and vice-versa (Pavan et al., 2005). Soil enzymes are touted as an index for examining the activities of microbes, soil productivity and soil quality owing to the symbiosis of microbes and flora (Dick, 1994; Bandick and Dick, 1999). Evaluation of diverse soil extracellular enzymes established it as a potent means for assessment of the soil functions for nutrient-cycling and microbial nutrient requirements (Sinsabaugh et al., 2012). We also hypothesized that in lieu of specific enzyme activity, an index merging diverse enzymes would be a more efficient and appropriate indicator of soil quality, since it could specify an inclusive diversity of soil functions. Relationships between crop yields, nutrient availability, and these enzyme activities are also obscure, as information on the enzymes’ ability to predict soil quality attributes is scarce. With the induction of intensive crop management practices like fertilizer application exhibiting complicated and harmful implications on plants and microbial associations, the studies on impact assessment of B-fertilization on soil microbes and enzymes for recuperating agricultural output in B-deficient soils become highly imperative (Dick, 1994; Tabatabai, 1994). The necessity of B-fertilization for the augmentation and maturity of plants has already been established (Shelp, 1993; Marschner, 1995). Boron acts as a vital function in the translocation and assimilation of complex carbohydrates in the plant, production of plant hormones and nucleic acids, germinating pollen, flower induction and fruiting, and water utilization. The main significant roles of B in plants are its structural role in cell wall growth and stimulating or inhibiting of precise metabolic pathways (Ahmad et al., 2009). In addition, B plays a crucial role in N assimilation, N fixation, and the growth of legume root nodules (Bolaños et al., 2004a; Bellaloui et al., 2014).

Boron accessibility depends on many criteria that exist in the soil–plant system such as SOM, soil texture, cultivation, soil moisture, temperature, soil pH and liming, soil fertility, and microbial activity (Shorrocks, 1997; Kumar et al., 2016; Shireen et al., 2018). Microbes assimilate SOM, which in turn, helps in releasing the B from organic complexes. Boron present in the soil is considered as a vital fraction related to SOM and is unleashed via microbial activities (Berger and Pratt, 1963). Despite the prime role of B on floral growth and functioning, no credibility has been established to explain that B is an enzyme component and possesses a direct role in enzyme actions. It is obscure to claim that these processes are precursor of the direct functioning of B or the indirect role of B. The biological effects of B are better understood in plants, where it has been proven that it can influence physiology and biological activities (Grattan et al., 2015). Though the impact of B on the soil microbial community is little known, there is a paucity of information on the element’s consolidative impacts on several characteristics of the soil microbial community, including activity, biomass, and diversity. Most of the soil fertility experiments stressed the aspects of the changes in soil chemical pools without giving much attention to biological attributes in soil rendering a lacuna on comprehensive fertility evaluation in soil fertility research. To date, the study on the role of B on soil biological attributes is obscure and very limited. Consequently, advanced research is necessitated to have a clear insight into the functioning of B in plant development and soil biology. The study hypothesized B-fertilization would improve the soil physio-chemical and biological properties and productivity of cauliflower-cowpea-okra cropping system. The objective of the study is to assess the impact of graded levels of B-fertilization on the soil physio-chemical and biological properties and their relationships in a cauliflower-cowpea-okra cropping system in North East India.

## Materials and methods

### Experimental site

**FIGURE 1:**
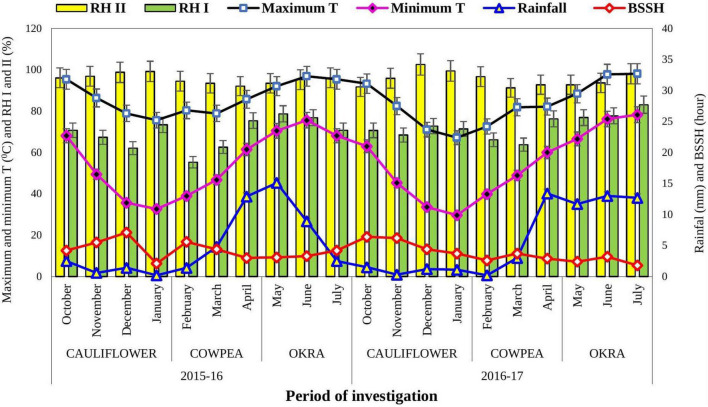
Meteorological data during the period of investigation for the year 2015–2017. Source: Department of Agrometeorology, Assam Agricultural University, Jorhat, Assam. The vertical bars represent the SE(m)±. T, temperature; RH, relative humidity; and BSSH, bright sunshine hour.

A field experiment was conducted on cauliflower-cowpea-okra cropping system at the Horticultural Experimental Farm, Assam Agricultural University, Jorhat, India (26°47′N latitude, 94°12′E longitude, 86.6 m altitude) during 2015–2017. The climate of the experimental site is sub-tropical with hot humid summers and comparatively dry and cool winters. Normal annual rainfall varies between 1,500 and 2,000 mm. Usually, rain commences from June and continues up to September with the pre-monsoon showers commencing from mid-March. The highest temperature of 34°C during summers and the lowest about 7°C during winters is usually prevalent. Agro-meteorological information is presented in Figure 1. The soil of the experimental site is Inceptisol having a sandy clay-loam texture with pH 4.8.

### Experimental design and treatments

The field experiments were uniformly laid-out for all the three crops CCOCS in a completely randomized block design with 4-replicates. Soil application of B was imposed at the rates of 0, 0.5, 1.0, 1.5 and 2.0 kg ha^–1^ in cauliflower, wherein its application was restricted only to cauliflower in both years of experimentation to assess the direct effect of B fertilization. However, B-fertilization was exempted in succeeding crops (cowpea and okra) to assess the residual effect of B-fertilization in the sequence. Borax (Na_2_B_4_O_7_.10H_2_O, analytical reagent grade with 10.5% B) was applied as the source of B for soil application. The 100% recommended dose of fertilizers (RDF) (supplied via urea, single super phosphate, and muriate of potash) and farmyard manure (FYM, well-decomposed cowdung) was uniformly applied to all the crops at the time of sowing (Supplementary Table 1). FYM used in the experiment had the bulk density of 0.24 Mg m^–3^, pH (7.7), N (1.4%), P (0.34%), K (0.8%), Mg (0.5%), Ca (1.4%), and C: N ratio (28:1) as determined using standard procedures (Rana et al., 2014).

### Soil sampling and analyses

For soil physio-chemical analysis, soil samples were collected from a depth of 0–15 cm at different crop growth stages in the sequence. While for soil biological properties, moist soil samples were collected at the initial and different crop growth stages of cauliflower, cowpea and okra for the two years. Soil samples were then stored in the refrigerator at 4°C for analysis of biological parameters. All the analyses were made in triplicate following standard protocols. The soil samples were analyzed for soil physico-chemical and biological properties (Supplementary Table 2).

The microbial quotient was calculated as the ratio of MBC to SOC and expressed in percentage (Anderson and Domsch, 1989).

#### Crop equivalent yields

After the harvest of each crop, the yield was recorded. Crop equivalent yield (CEY) was computed to evaluate system performance after converting the yield of one crop (assumed as x) into the equivalent yield of another crop (assumed as y) on a pricing basis:


$$EY_{X}=Y_{X}\frac{P_{X}}{P_{Y}}$$

Where, EY_*x*_ = yield of x crop converted to yield of y crop, Y_*x*_ is the yield of x crops (kg ha^–1^), P_*x*_ is the price of x crops (US$ kg^–1^), and P_*y*_ is the price of y crop (US$ kg^–1^). All the yields of crops were converted to the equivalent yield of cauliflower, which was planted first in the cropping sequence.

### Enzyme activity-based index for calculation of soil functional diversity

#### Soil quality index

##### Geometric mean of enzymatic activities

To better explicate the impact of B on soil enzyme activities, we computed the geometric mean of enzymatic activities (GMEA), as it can replicate the inclusive enzyme activity levels (Hinojosa et al., 2004). GMEA is a consolidative method to pool the enzyme activities associated to diverse soil functions and nutrients; therefore, possibly it will reflect soil quality index (Paz-Ferreiro et al., 2012).

GMEA of the assayed enzymes was computed for each sample as:


$$\text{GMEA}={\left(\text{FDA}\times \text{PMEase}\times \text{DH}\times \text{AS}\times \text{UE}\right)}^{1/5}$$

#### Soil functional diversity

The Shannon index and Simpson-Yule index cater the data about the spread or distribution of C source usage by the microbial community (Kumar et al., 2017). The ensuing indexes can be used for quantification of richness, evenness, and diversity of the soil microbial community.

It was computed using the following equations

Shannon’s diversity index (H)


$$\text{H}=-\sum_{i=1}^{5}Pi\times \ln\left(Pi\right)$$

where, Pi is the ratio of each enzyme activity to the summation of whole enzymes activities for a specific sample. Enzyme activities were deciphered as μg product formed per g of soil per hour.

Simpson-Yule index (SYI)

SYI was calculated as for each sample as:


$$\text{SYI}=\frac{1}{\sum_{i=1}^{5}{\text{Pi}}^{2}}$$

The diversity of the community is directly proportional to H.

### Multivariate analysis

#### Hierarchical cluster analysis

The data obtained on the different groups of biological entities present in the soil samples were subjected to agglomerative hierarchical cluster analysis (HCA) (Euclidean distances, Ward’s agglomeration rule) to establish homogeneous groupings of data. The nodes depicted clusters retrieved on each step of hierarchical clustering.

#### Principal component analysis

The principal component analysis (PCA) of all the data was performed (Andrewsi and Carroll, 2001; Andrews et al., 2002) to ascertain the variability and show the relationship among the various soil properties, and to extract the dominant principal components from the whole data set in soil resorting to R studio. PCA is a multivariate statistical dimension reduction tool that resorts to an orthogonal transformation to transform a set of correlated variables to linearly uncorrelated variables known as principal components (PC). The extracted results of a PCA are displayed in terms of component scores, also called factor scores and loadings (Wold et al., 1987). In the PCA algorithm, diminution of the number of components was yielded via the eigenvalue-one criterion i.e., eigenvalue >1 is retained, also called as Kaiser criterion (Kaiser, 1960), and the scree test (Cattell, 1966).

#### Stepwise multiple linear regression analysis

Stepwise multiple linear regression analyses were performed using R studio with the backward exclusion method to explore the significance of dominant soil biological entities in the prediction of crop yield in the sequence. The relationship between a single response variable (dependent variable) and two or more controlled variables was evaluated using multiple stepwise linear regressions (MLR) (independent variables). MLR used in the research states that the higher R^2^ generates good results in model fitting (Bowerman et al., 2005). The analytical model, used to develop a model for predicting crop yield from the biological attribute’s relationship is given by the equation:


$$\text{Y}=\beta_{0}+\beta_{1}{\text{X}}_{1}+\beta_{2}{\text{X}}_{2}+\ldots +\beta_{k}{\text{X}}_{k}+\varepsilon$$

Where, ***βs*** are coefficients, ***Xi*** are the predictors, ***Y*** is the crop yield (response) and ***β*_0_** is a constant.

The null hypothesis of sequential uncorrelated errors was tested independently on regression residuals using the Durbin–Watson statistic.

#### Path analysis (causal modeling)

Path analysis is a standardized partial regression analysis used to determine the significance of the relationship between sets of variables and to provide estimates of the magnitude to make the multiple regressions easier to comprehend. It also helps to figure out the direct, indirect and total impact of predictor variables on the response variable. An evaluation of correlation does not specify the precise influence of the attributes to crop yield and this correlation can be segregated into direct and indirect effects via path coefficient analysis. It permits the separation of the direct and their indirect effects via other traits through allocating the correlations (Wright, 1921) for clarity of explanation of cause and effect.

### Data analysis and visualization

The experimental data obtained from different observations were analyzed statistically by using Fisher’s method of ANOVA in randomized block design (Panse and Sukhatme, 1985). Significance or non-significance of the variance due to different treatment effects was estimated by computing concerned ‘F’ values. At a 95% confidence level, the experimental means were compared. To compare treatment means, the Duncan Multiple-Range-Test (DMRT) was employed. Univariate Pearson’s correlation analysis was executed to determine the interrelationship between biological entities in the soil samples and crop yield. A correlograms was built using the “*corrgram package”* in R Studio.

## Results

### Crop yield and cauliflower-equivalent-yield

**FIGURE 2:**
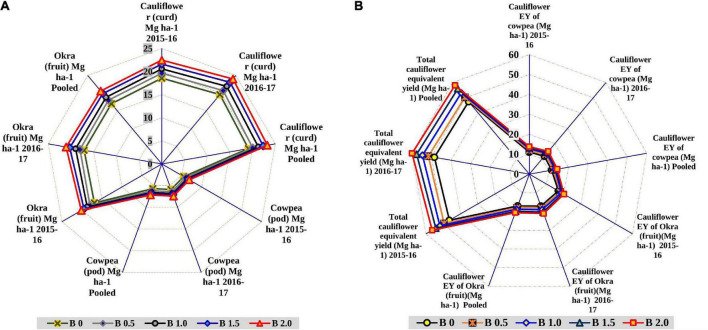
Effect of graded B levels on crop yield (Mg ha^–1^) in cauliflower-cowpea–okra cropping system **(A)**; effect of graded B levels on CEY (Mg ha^–1^) in cauliflower-cowpea–okra cropping system **(B)**.

In general, the crop yields in the cauliflower-cowpea-okra cropping system were significantly impacted by the imposition of a graded level of B, as evident by the significant augmentation in yield (Figure 2A). A satisfactory cauliflower curd yield (highest) of 23.25 Mg ha^–1^ was obtained as a ramification of the direct effect of 2 kg B ha^–1^ imposition in cauliflower with drastic yield augmentation up to 21.8% over the control (19.09 Mg ha^–1^). While the residual implication of 2 kg B ha^–1^ in cowpea and okra, also leveraged the pod yield to the tune of 7.15 Mg ha^–1^ and fruit yield of 20.61 Mg ha^–1^, thereby, improving the crop yield by 25.7% over the control (5.69 Mg ha^–1^) in cowpea and 21.2% over the control in okra (17.0 Mg ha^–1^). Likewise, the CEY of the cauliflower-cowpea-okra cropping system was significantly greater (*p* < 0.05) with an imposition of 2 kg B ha^–1^ as compared to control (Figure 2B). The extent of growth in equivalent yield was 25.7, 21.3, and 22.5% for CEY of cowpea, CEY of okra and total CEY, respectively over control. The increase was always higher with 2 kg B ha^–1^ than the rest of the B levels including control.

### Soil physico-chemical properties

Imposition of differential B levels could not significantly (*p* ≤ 0.05) affect the soil BD under cauliflower-cowpea-okra cropping system (Table 1), however, a decrease in BD was noticed in B applied plots as compared to control (B_0_). In general, the experimental soils were mostly acidic in reaction with pH 4.83–4.89 (Table 1), with no significant difference among the B treatments. Continuous two years of experimentation decreased the BD in soil by 2.4% as compared to the initial status on addition of a maximum B level of 2 kg ha^–1^. However, improvements in soil pH and SOC were noticed to the tune of 1.5 and 30% over the initial status when 2 kg B ha^–1^ was applied. The available N and P (Table 1), were also significantly (*p* ≤ 0.05) influenced by the addition of graded B levels wherein the greatest value was always with 2 kg B ha^–1^ application.

**TABLE 1:** Effect of graded B levels on soil physico-chemical properties at different crop stages in cauliflower-cowpea-okra cropping system (Pooled data 2015–017).

B-levels (kg ha^–1^)	Bulk density (BD)			Soil pH			Soil organic carbon (%)			Available N			Available P		
	Vegetative stage														
	Cauliflower	Cowpea	Okra	Cauliflower	Cowpea	Okra	Cauliflower	Cowpea	Okra	Cauliflower	Cowpea	Okra	Cauliflower	Cowpea	Okra
B_0_	1.24^a^	1.23^a^	1.22^a^	4.83a	4.87a	4.82a	0.90a	0.95a	0.91a	301.20d	311.50d	304.73d	15.95c	18.13c	20.31c
B_0.5_	1.23^a^	1.22^a^	1.21^a^	4.85a	4.88a	4.85a	0.95a	1.01a	0.94a	307.25c	318.65c	310.95c	17.00bc	19.50bc	21.33bc
B_1.0_	1.24^a^	1.23^a^	1.21a	4.87a	4.88a	4.86a	0.99a	1.01a	0.97a	314.91bc	324.6b	317.61bc	17.98ab	20.58b	22.45ab
B_1.5_	1.23^a^	1.22^a^	1.22a	4.86a	4.89a	4.85a	1.01a	1.02a	0.99a	322.56b	329.95b	324.76b	18.95ab	21.55b	23.41ab
B_2.0_	1.23^a^	1.22^a^	1.21a	4.86a	4.89a	4.85a	1.01a	1.04a	1.00a	328.06a	335.35a	329.83a	20.03a	22.63a	24.70a
Mean	1.23	1.22	1.21	4.85	4.88	4.85	0.97	1.01	0.96	314.80	324.01	317.58	17.98	20.48	22.44

	Reproductive stage in different crops*														
	**Cauliflower**	**Cowpea**	**Okra**	**Cauliflower**	**Cowpea**	**Okra**	**Cauliflower**	**Cowpea**	**Okra**	**Cauliflower**	**Cowpea**	**Okra**	**Cauliflower**	**Cowpea**	**Okra**
	**Cis**	**Fls**	**Frs**	**Cis**	**Fls**	**Frs**	**Cis**	**Fls**	**Frs**	**Cis**	**Fls**	**Frs**	**Cis**	**Fls**	**Frs**

B_0_	1.24^a^	1.23a	1.22a	4.81a	4.86a	4.84a	0.86a	0.92a	0.89a	301.30d	307.33cd	302.63c	15.63c	15.49c	18.98c
B_0.5_	1.23^a^	1.23a	1.21a	4.84a	4.87a	4.85a	0.91a	0.94a	0.92a	306.85bc	313.20c	308.55bc	16.59bc	16.50bc	20.02bc
B_1.0_	1.24^a^	1.23a	1.21a	4.84a	4.87a	4.85a	0.95a	0.96a	0.93a	313.51bc	318.37ab	315.31bc	17.54ab	17.57b	20.99bc
B_1.5_	1.24a	1.23a	1.21a	4.85a	4.87a	4.85a	0.98a	0.97a	0.95a	319.66b	323.55ab	321.20b	18.52ab	18.62b	22.00b
B_2.0_	1.23a	1.22a	1.21a	4.85a	4.86a	4.86a	1.02a	0.97a	0.95a	324.87a	328.89a	326.17a	19.45a	19.65a	23.07a
Mean	1.24	1.23	1.21	4.84	4.87	4.85	0.94	0.95	0.93	313.24	318.27	253.06	17.55	17.57	21.01

	**Maturity stage**														
	**Cauliflower**	**Cowpea**	**Okra**	**Cauliflower**	**Cowpea**	**Okra**	**Cauliflower**	**Cowpea**	**Okra**	**Cauliflower**	**Cowpea**	**Okra**	**Cauliflower**	**Cowpea**	**Okra**

B_0_	1.24a	1.24a	1.22a	4.82a	4.85a	4.86a	0.82a	0.93a	0.91a	296.40d	300.82d	299.10c	14.89c	15.37c	17.65c
B_0.5_	1.23a	1.22a	1.21a	4.83a	4.85a	4.87a	0.84a	0.95a	0.92a	302.00c	307.03cd	304.70bc	15.97ab	16.52bc	18.72b
B_1.0_	1.23a	1.23a	1.22a	4.83a	4.86a	4.87a	0.85a	0.95a	0.93a	308.11bc	312.53c	310.81bc	16.94ab	17.44ab	19.73b
B_1.5_	1.22a	1.23a	1.21a	4.84a	4.86a	4.87a	0.85a	0.96a	0.93a	312.76b	317.54b	315.46b	17.87a	18.39ab	20.69ab
B_2.0_	1.22a	1.23a	1.21a	4.84a	4.86a	4.88a	0.86a	0.97a	0.94a	317.56a	323.01a	320.26a	18.81a	19.41a	21.84a
Mean	1.23	1.23	1.21	4.83	4.86	4.87	0.84	0.95	0.93	307.37	312.19	310.07	16.90	17.43	19.73

*Cis, curd initiation stage in cauliflower; Fls, flowering stage in cowpea; Frs, fruiting stage in okra. The means followed by a different letter are significantly different at *p* ≤ 0.05 by Duncan’s multiple range test (values are means of 3-replicates).

### Implication of B on temporal dynamics in microbiological pools of soil organic matter

The soil microbial biomass carbon (MBC), Microbial quotient (MBC: SOC) microbial biomass-N, (MBN), Microbial biomass-P (MBP), and soil respiration (SR) improved significantly (*p* ≤ 0.05) on the application of different B doses at all the stages of crop growth over the control with few exceptions in case of MBN (Table 2). The highest values of MBC, MBC: SOC MBN, MBP, and SR in all the crop growth stages in different crops were with 2.0 kg B ha^–1^ addition. After 2-years of experimentation, there was a gain of 24.5, 12.1, 54.2, 34.4, and 36.1% in MBC, MBC: SOC MBN, MBP, and SR over the initial soil status. The order of the B treatments in respect of MBC, MBN, and SR at different crop growth stages was 2.0 kg B ha^–1^ > 1.5 B kg ha^–1^ > 1.0 B kg ha^–1^ > 0.5 B kg ha^–1^> 0 kg B ha^–1^ (Control), respectively.

**TABLE 2:** Effect of graded B levels on microbiological pools of soil organic matter at different crop stages in cauliflower-cowpea-okra cropping system (Pooled data 2015–2017).

B-levels (kg ha^–^^1^)	Microbial biomass-C (MBC) (μg g^–,1^)			Microbial quotient (MBC: SOC)			Microbial biomass-N (MBN) (μg g^–1^)			Microbial biomass-P (MBP) (μg g^–1^)			Soil respiration (SR) (μg CO_2_ day^–1^ g^–1^ FW)		
	Vegetative stage														
	Cauliflower	Cowpea	Okra	Cauliflower	Cowpea	Okra	Cauliflower	Cowpea	Okra	Cauliflower	Cowpea	Okra	Cauliflower	Cowpea	Okra
B_0_	175.35^c^	179.76^d^	176.16^c^	19.48^c^	18.92^bc^	19.36^c^	41.84^b^	48.97^a^	52.82^b^	4.42d	4.62c	4.57d	6.44^c^	6.30^d^	6.78^c^
B_0.5_	189.11^ab^	184.36^c^	180.91^bc^	19.91^b^	18.25^bc^	19.25^ab^	41.99^a^	49.21^a^	53.17^a^	4.86c	4.88bc	4.74c	6.56^bc^	6.41^bc^	6.91^bc^
B_1.0_	195.83^ab^	191.72^c^	186.17^ab^	19.78^ab^	18.98^b^	19.19^ab^	42.21^a^	49.11^a^	53.74^a^	4.91bc	4.95b	4.79b	6.62^ab^	6.49^bc^	6.99^b^
B_1.5_	200.68^b^	208.16^b^	191.29^ab^	19.87^a^	20.41^b^	19.32^a^	42.89^a^	49.98^a^	53.93^a^	4.96b	5.02ab	4.84b	6.69^ab^	6.59^b^	7.05^b^
B_2.0_	205.83^a^	214.81^a^	195.68^a^	20.38^a^	20.65^a^	19.57^a^	43.42^a^	51.07^a^	54.01^a^	5.01a	5.08a	4.92a	6.77^a^	6.71^a^	7.12^a^
Mean	193.36	195.76	186.04	19.88	19.44	19.34	32.47	39.67	43.53	4.83	4.91	4.77	6.62	6.50	6.97

	Reproductive stage in different crops*														
	**Cauliflower**	**Cowpea**	**Okra**	**Cauliflower**	**Cowpea**	**Okra**	**Cauliflower**	**Cowpea**	**Okra**	**Cauliflower**	**Cowpea**	**Okra**	**Cauliflower**	**Cowpea**	**Okra**
	**Cis**	**Fls**	**Frs**	**Cis**	**Fls**	**Frs**	**Cis**	**Fls**	**Frs**	**Cis**	**Fls**	**Frs**	**Cis**	**Fls**	**Frs**

B_0_	179.94^d^	182.85^d^	176.01^d^	20.92^bc^	19.88^c^	19.78^c^	50.67^b^	58.03^b^	54.67^a^	4.68d	4.74d	4.65c	6.47^c^	7.04^c^	6.64^c^
B_0.5_	191.44^c^	194.34^bc^	189.27^bc^	21.04^bc^	20.67^bc^	20.57^b^	50.94^a^	58.44^a^	54.73^a^	4.87c	4.91c	4.82bc	6.60^bc^	7.15^b^	6.77^b^
B_1.0_	194.95^c^	199.86^b^	194.17^bc^	20.52^b^	20.82^bc^	20.88^ab^	51.11^a^	58.61^a^	54.82^a^	4.92b	4.95bc	4.86b	6.68^b^	7.23^b^	6.85^ab^
B_1.5_	199.75^b^	204.90^b^	200.27^b^	20.38^b^	21.12^b^	21.08^ab^	51.15^a^	58.98^a^	54.91^a^	4.96b	5.01b	4.90b	6.85^b^	7.31^ab^	6.94^ab^
B_2.0_	206.19^a^	209.93^a^	205.88^a^	20.21^a^	21.64^a^	21.67^a^	51.89^a^	59.23^a^	55.06^a^	5.01a	5.06a	4.95a	6.88^a^	7.42^a^	7.02^a^
Mean	194.45	198.38	193.12	20.62	20.83	20.80	51.15	58.66	54.84	4.89	4.93	4.84	6.70	7.23	6.84

	**Maturity stage**														
	**Cauliflower**	**Cowpea**	**Okra**	**Cauliflower**	**Cowpea**	**Okra**	**Cauliflower**	**Cowpea**	**Okra**	**Cauliflower**	**Cowpea**	**Okra**	**Cauliflower**	**Cowpea**	**Okra**

B_0_	181.57^d^	188.74^d^	178.61^c^	22.14^c^	20.29^bc^	19.63^c^	43.87^b^	57.21^b^	55.95^a^	4.51c	4.64c	4.48d	6.64^c^	6.88^c^	6.84^d^
B_0.5_	192.87^c^	195.05^bc^	187.20^bc^	22.96^b^	20.53^b^	20.35^ab^	44.22^a^	57.92^a^	56.68^a^	4.72bc	4.81b	4.64c	6.77^b^	6.99^bc^	6.97^c^
B_1.0_	197.42^bc^	201.19^bc^	194.71^b^	23.23^a^	21.18^ab^	20.94^b^	44.31^a^	57.98^a^	56.72^a^	4.76b	4.85ab	4.68c	6.85^ab^	7.07^b^	7.05^b^
B_1.5_	203.56^b^	205.96^b^	199.70^ab^	23.95^a^	21.45^ab^	21.47^a^	44.32^a^	58.04^a^	56.74^a^	4.84ab	4.89a	4.73b	6.94^ab^	7.15^b^	7.14^b^
B_2.0_	208.60^a^	211.94^a^	205.25^a^	24.26^a^	21.85^a^	21.84^a^	44.47^a^	58.11^a^	56.82^a^	4.88a	5.94a	4.78a	7.02^a^	7.24^a^	7.22^a^
Mean	196.80	200.58	193.09	23.31	21.06	20.84	44.24	57.85	56.58	4.74	5.03	4.66	6.84	7.07	7.04

*Cis, curd initiation stage in cauliflower; Fls, flowering stage in cowpea; Frs, fruiting stage in okra. The means followed by a different letter are significantly different at *p* ≤ 0.05 by Duncan’s multiple range test (values are means of 3-replicates).

### Potentially mineralizable-C and potentially mineralizable-N

A significant (*p* ≤ 0.05) increasing trend in potentially mineralizable-C (PMC) and PMN with the B application rate was noted in the soil of CCOCS (Table 3). Comparatively, the higher recoveries of PMC and PMN were always higher with 2.0 kg B ha^–1^ direct and residual impact of B-fertilization in CCOCS at all the crop growth stages. The PMC and PMN across the crops and growth phases in the sequence were ranked as 2 kg B ha^–1^> 1.5 B kg ha^–1^> 1.0 B kg ha^–1^> 0.5 B kg ha^–1^> 0.0 kg B ha^–1^ (Control). Compared with the initial soil status, there was an improvement of 26.3 and 52.5% in respect of PMC and PMN content in soil due to the imposition of graded levels of B.

**TABLE 3:** Effect of graded B levels on PMN and PMC content in soil at different crop stages of cauliflower-cowpea-okra cropping system.

B-levels (kg ha^–1^)	Potentially mineralisable-C			Potentially mineralisable-N		
	Vegetative stage					
	Cauliflower	Cowpea	Okra	Cauliflower	Cowpea	Okra
B_0_	144.35^c^	148.76^c^	140.16^c^	29.35**^c^**	32.76^bc^	30.16^c^
B_0.5_	151.11^bc^	153.36^bc^	146.91^bc^	31.11^b^	35.36^bc^	34.91^bc^
B_1.0_	155.83^b^	160.72^ab^	152.17^ab^	32.76^ab^	36.72^b^	36.17^bc^
B_1.5_	159.68^ab^	167.16^b^	160.29^b^	34.68^ab^	38.16^b^	37.29^b^
B_2.0_	164.83^a^	173.81^a^	164.68^a^	35.83a	40.01^a^	38.68^a^
Mean	155.16	160.76	152.84	30.15	37.56	46.44

	Reproductive stage in different crops*					
	**Cauliflower**	**Cowpea**	**Okra**	**Cauliflower**	**Cowpea**	**Okra**
	**Cis**	**Fls**	**Frs**	**Cis**	**Fls**	**Frs**

B_0_	152.94^c^	151.85^c^	145.01^c^	29.94^c^	34.85^c^	35.01^c^
B_0.5_	158.44^b^	157.34^bc^	158.27^b^	30.44^c^	36.34^bc^	37.27^bc^
B_1.0_	161.95^b^	160.86^bc^	163.17^b^	31.95^ab^	37.86^bc^	38.17^b^
B_1.5_	165.75^b^	163.9^b^	166.27^b^	33.75^ab^	38.39^b^	39.27^b^
B_2.0_	168.19^a^	167.93^a^	171.88^a^	35.19^a^	39.93^a^	30.88^a^
Mean	171.45	162.38	160.92	41.45	59.98	63.12

	**Maturity stage**					
	**Cauliflower**	**Cowpea**	**Okra**	**Cauliflower**	**Cowpea**	**Okra**

B_0_	154.57^c^	152.74^c^	147.61^c^	28.57^c^	37.74^c^	36.61^c^
B_0.5_	160.87^bc^	160.05^bc^	152.2^b^	32.87^b^	38.05^bc^	37.20^b^
B_1.0_	164.66^bc^	165.19^bc^	158.71^ab^	34.66^b^	39.19^bc^	38.71^ab^
B_1.5_	168.98^b^	169.96^b^	163.70^ab^	35.98^b^	40.96^b^	39.70^ab^
B_2.0_	171.76^a^	174.94^a^	168.25^a^	37.76^a^	41.24^a^	40.25^a^
Mean	171.17	187.78	158.09	71.17	84.18	88.09

*Cis, curd initiation stage in cauliflower; Fls, flowering stage in cowpea; Frs, fruiting stage in okr. The means followed by a different letter are significantly different at *p* ≤ 0.05 by Duncan’s multiple range test (values are means of 3-replicates).

#### Microbial populations

There was significant (*p* ≤ 0.05) improvement in microbial populations (actinomycetes, bacteria and fungi) across B treatments in all crops in the sequence (Table 4). The addition of escalated B level of 2.0 kg B ha^–1^ led to a significant improvement in the status of microbial populations in the soil at different crop growth stages in cauliflower (direct application of B); and that in cowpea and okra (residual effect of B). Interestingly, this escalated B treatment led to an augmentation of 54.2, 55.3 and 53.7% of actinomycetes, bacteria and fungi population in comparison to the initial soil status.

**TABLE 4:** Effect of graded B levels on microbial populations in soil under cauliflower-cowpea-okra cropping system (Pooled data 2015–2017).

B-levels (kg ha^–1^)	Total actinomycetes population (cfu g^–1^ soil)			Total bacterial population (cfu g^–1^ soil)			Total fungal population (cfu g^–1^ soil)		
	Vegetative stage								
	Cauliflower	Cowpea	Okra	Cauliflower	Cowpea	Okra	Cauliflower	Cowpea	Okra
B_0_	16.44^c^	18.51^c^	17.18^d^	7.30^c^	8.59^c^	7.49^c^	14.14^c^	16.81^c^	17.20^bc^
B_0.5_	17.56^bc^	19.62^bc^	18.31^c^	7.97^bc^	9.83^bc^	8.27^bc^	14.50^b^	17.02^b^	17.52^b^
B_1.0_	18.62^b^	20.71^b^	19.39^ab^	8.15^b^	10.80^bc^	8.47^b^	15.10^ab^	17.31^ab^	17.71^b^
B_1.5_	19.69^ab^	21.79^b^	20.45^ab^	8.31^ab^	11.79^ab^	8.65^ab^	15.41^ab^	17.53^ab^	17.92^ab^
B_2.0_	20.77^a^	22.88^a^	21.52^a^	8.74^a^	12.84^a^	8.84^a^	15.82^a^	17.72^a^	18.30^a^
Mean	18.62	20.70	19.37	8.69	10.77	10.52	16.73	17.45	17.73

	Reproductive stage in different crops*								
	**Cauliflower**	**Cowpea**	**Okra**	**Cauliflower**	**Cowpea**	**Okra**	**Cauliflower**	**Cowpea**	**Okra**
	**Cis**	**Fls**	**Frs**	**Cis**	**Fls**	**Frs**	**Cis**	**Fls**	**Frs**

B_0_	16.47^c^	19.64^c^	17.44^c^	7.38^c^	9.01^c^	8.57^c^	14.09^c^	19.61^c^	17.33^c^
B_0.5_	17.61^b^	20.77^ab^	18.55^bc^	8.04^bc^	10.08^bc^	9.04^bc^	16.41^b^	20.12^b^	17.51^b^
B_1.0_	18.68^ab^	21.85^ab^	19.63^bc^	8.31^bc^	10.30^b^	9.27^b^	16.70^b^	20.36^ab^	17.72^b^
B_1.5_	19.8^ab^	22.94^a^	20.71^ab^	8.63^b^	10.61^b^	9.45^ab^	17.02^b^	20.52^ab^	17.90^ab^
B_2.0_	20.88^a^	23.02^a^	21.82^a^	8.94^a^	10.87^a^	9.67^a^	17.31^a^	20.75^a^	18.14^a^
Mean	18.69	21.64	19.63	9.44	11.37	11.18	16.48	20.31	17.72

	**Maturity stage**								
	**Cauliflower**	**Cowpea**	**Okra**	**Cauliflower**	**Cowpea**	**Okra**	**Cauliflower**	**Cowpea**	**Okra**

B_0_	16.3^c^	18.88^c^	17.24^d^	7.41^c^	9.78^c^	9.68^c^	15.12^c^	20.17^c^	17.72^b^
B_0.5_	17.41^bc^	19.99^bc^	18.37^c^	8.15^b^	10.13^bc^	10.01^b^	16.85^bc^	20.82^bc^	18.0^ab^
B_1.0_	18.49^b^	20.07^bc^	19.45^bc^	8.59^ab^	10.24^ab^	10.21^ab^	17.13^ab^	21.01^ab^	18.21^ab^
B_1.5_	19.59^ab^	21.15^b^	20.54^b^	9.81^b^	10.47^ab^	10.42^b^	17.32^ab^	21.25^a^	18.42^ab^
B_2.0_	20.70^a^	22.24^a^	21.62^a^	10.10^a^	10.68^a^	10.67^a^	17.65^a^	21.42^a^	18.71^a^
Mean	20.47	19.44	9.67	11.88	11.84	16.81	20.99	18.21	20.47

*Cis, curd initiation stage in cauliflower; Fls, flowering stage in cowpea; Frs, fruiting stage in okra. The means followed by a different letter are significantly different at *p* ≤ 0.05 by Duncan’s multiple range test (values are means of 3-replicates).

### Soil enzymes

Soil enzymes *viz.*, AS, DH, FDA, and PMA showed a significant (*p* ≤ 0.05) response to graded B levels in soil (Table 5). However, the urease enzyme (UE) showed an antagonistic effect, thereby, exhibiting a reciprocal response to the appliance of the graded levels of B. Across all crop growth phases in the sequence, higher enzyme activities were noticed under plots receiving higher B levels of 2.0 kg ha^–1^. The status of the content of soil enzymes in diverse crops and growth stages varied as: 2.0 kg B ha^–1^> 1.5 B kg ha^–1^> 1.0 B kg ha^–1^> 0.5 B kg ha^–1^> 0.0 kg B ha^–1^. Soil enzymes’ activity registered an increment to the tune of 44.7 (AS), 45.1 DH), 38.6 (FDA) and 46.7% (PMA), respectively over the initial soil status. On contrary to that, UE enzyme activity exhibited a decrement by 20.7% over the initial soil status.

**TABLE 5:** Effect of graded B levels on soil enzymes in cauliflower-cowpea-okra cropping system (Pooled data 2015–2017).

B-levels (kg ha^–1^)	Fluorescein di-acetate (FDA) hydrolysis activity (μg fluorescein g^–1^ h^–1^)			Phosphomonoesterase (PMEase) activity (μgp-nitrophenol g^–1^ h^–1^)			Dehydrogenase (DH) activity (μg TPF g^–1^ 24 h^–1^)			Arylsulphatase (AS) activity (μgp-nitrophenol g^–1^ h^–1^)			Urease activity (UE) (μg NH_4_-N g^–1^ soil 2 h^–1^)		
	Vegetative stage														
	Cauliflower	Cowpea	Okra	Cauliflower	Cowpea	Okra	Cauliflower	Cowpea	Okra	Cauliflower	Cowpea	Okra	Cauliflower	Cowpea	Okra
B_0_	5.11^b^	5.21^c^	5.02^bc^	42.34^d^	44.47^c^	43.25^c^	122.21^cd^	135.96^c^	117.21^c^	8.15^b^	8.41^c^	8.13^c^	29.65^a^	24.24^a^	21.24^a^
B_0.5_	5.71^b^	5.85^bc^	5.78^bc^	44.59^c^	46.51^b^	45.29^bc^	131.45^c^	146.59^b^	126.45^bc^	9.42^b^	9.03^b^	8.84^bc^	28.76^ab^	22.95^a^	19.85^b^
B_1.0_	6.24^ab^	6.54^bc^	6.29^b^	46.22^b^	48.16^ab^	46.94^b^	140.11^bc^	151.81^b^	135.11^bc^	9.71^ab^	9.32^ab^	9.22^b^	27.91^ab^	21.07^ab^	18.57^b^
B_1.5_	6.79^a^	6.93^b^	6.64^a^	47.94^b^	49.66^a^	48.44^b^	146.08^b^	159.28^ab^	141.08^b^	10.02^a^	9.51^ab^	9.59^b^	26.54^b^	19.91^bc^	18.21^b^
B_2.0_	7.12^a^	7.29^a^	6.99^a^	49.12^a^	50.87^a^	49.65^a^	152.25^a^	166.95^a^	146.75^a^	10.41^a^	9.74^a^	9.74^a^	24.43^c^	19.78^c^	17.28^c^
Mean	6.19	6.36	6.14	46.04	47.93	46.71	138.42	152.12	133.32	11.14	13.14	14.30	27.46	21.03	18.23

	Reproductive stage in different crops*														
	**Cauliflower**	**Cowpea**	**Okra**	**Cauliflower**	**Cowpea**	**Okra**	**Cauliflower**	**Cowpea**	**Okra**	**Cauliflower**	**Cowpea**	**Okra**	**Cauliflower**	**Cowpea**	**Okra**
	**Cis**	**Fls**	**Frs**	**Cis**	**Fls**	**Frs**	**Cis**	**Fls**	**Frs**	**Cis**	**Fls**	**Frs**	**Cis**	**Fls**	**Frs**

B_0_	5.22^c^	6.06^c^	5.17^c^	43.89^c^	45.24^c^	44.02^c^	129.32^d^	138.66^c^	124.51^c^	9.13^c^	9.71^c^	9.10^bc^	31.25^a^	22.22^a^	27.22^a^
B_0.5_	5.89^bc^	6.69^bc^	5.67^bc^	45.65^ab^	47.71^b^	46.49^bc^	138.54^c^	147.74^bc^	134.25^b^	10.41^b^	10.22^b^	9.38^b^	29.98^b^	20.94^ab^	25.54^ab^
B_1.0_	6.34^b^	7.06^ab^	6.05^ab^	47.52^ab^	49.47^b^	48.25^b^	147.11^bc^	156.08^b^	142.91^ab^	10.74^b^	10.44^ab^	9.53^ab^	28.71^b^	21.05^b^	24.45^ab^
B_1.5_	6.79^b^	7.36^ab^	6.49^ab^	49.26^ab^	50.87^ab^	49.65^b^	152.86^bc^	164.88^ab^	149.38^ab^	10.92^b^	10.68^ab^	9.67^ab^	27.20^b^	19.97^b^	23.39^ab^
B_2.0_	7.04^a^	7.81^a^	6.75^a^	50.32^a^	52.05^a^	50.83^a^	160.75^a^	171.55^a^	155.05^a^	11.22^a^	10.93^a^	9.84^a^	21.61^c^	18.84^c^	22.24^c^
Mean	6.42	7.14	6.35	47.33	49.07	47.85	145.72	155.78	141.22	11.90	15.26	14.38	37.75	39.00	23.97

	**Maturity stage**														
	**Cauliflower**	**Cowpea**	**Okra**	**Cauliflower**	**Cowpea**	**Okra**	**Cauliflower**	**Cowpea**	**Okra**	**Cauliflower**	**Cowpea**	**Okra**	**Cauliflower**	**Cowpea**	**Okra**

B_0_	5.41^c^	6.30^c^	5.48^c^	44.67^c^	45.93^c^	44.71^c^	135.59^c^	142.01^c^	127.41^c^	10.04^c^	10.36^c^	9.67^c^	28.08^a^	23.67^a^	26.85^a^
B_0.5_	5.95^b^	6.84^c^	5.84^bc^	46.96^ab^	48.4^b^	47.18^b^	144.15^bc^	150.75^bc^	136.65^bc^	10.31^bc^	10.83^bc^	10.03^b^	27.12^ab^	22.05^a^	26.74^ab^
B_1.0_	6.24^ab^	7.02^bc^	6.16^bc^	48.73^ab^	50.4^ab^	49.18^b^	151.31^bc^	157.91^b^	145.31^b^	10.43^b^	11.04^bc^	10.38^b^	25.90^ab^	21.95^b^	25.01^b^
B_1.5_	6.41^ab^	7.34^b^	6.40^b^	50.11^a^	53.85^a^	50.93^ab^	157.28^b^	161.36^b^	151.28^b^	10.64^b^	11.26^b^	10.57^b^	24.81^ab^	20.54^b^	23.45^b^
B_2.0_	6.70^a^	7.58^a^	6.68^a^	51.18^a^	54.10^a^	52.41^a^	164.95^a^	168.05^a^	156.95^a^	10.89^a^	11.41^a^	10.79^a^	22.32^c^	19.26^c^	22.98^c^
Mean	5.41	6.3	5.48	44.67	45.93	44.71	135.59	142.01	127.41	12.24	15.36	14.67	28.8	33.67	17.85

*Cis, Curd initiation stage in cauliflower, Fls, flowering stage in cowpea, Frs, fruiting stage in okra. The means followed by a different letter are significantly different at *p* ≤ 0.05 by Duncan’s multiple range test (values are means of 3-replicates).

### Soil quality index and functional diversity index

The GMEA was significantly (*p* ≤ 0.05) higher under plots receiving the highest B levels of 2 kg ha^–1^ at all the crop growth stages under CCOCS (Table 6). Irrespective of crop growth stages, direct application of 2 kg B ha^–1^ in cauliflower recorded comparatively higher GMEA than that under cowpea and okra (residual effect), respectively (Table 6). Similarly, the functional diversity indexes (H and SYI) exhibited the similar trend wherein their higher values were observed under plots receiving 2 kg B ha^–1^ (Table 6).

**TABLE 6:** Geometric mean enzyme activity (GMEA), Shannon diversity index (H) and Simpson diversity index (SYI) as affected by graded B levels under cauliflower-cowpea-okra cropping system (Pooled data 2015–2017).

B-levels (kg ha^–1^)	Geometric mean of enzymatic activities (GMEA)			Shannon diversity index (H)			Simpson diversity index (SYI)		
	Cauli-flower	Cowpea	Okra	Cauli-flower	Cowpea	Okra	Cauli-flower	Cowpea	Okra
	Vegetative stage								
B_0_	22.97^c^	22.99^c^	21.31^bc^	2.35bc	2.24c	2.22c	3.76^c^	3.69^bc^	3.44^c^
B_0.5_	24.63^b^	24.18^b^	22.53^bc^	2.36bc	2.26ab	2.24c	3.79^bc^	3.71^bc^	3.48^c^
B_1.0_	25.58^ab^	24.80^b^	23.27^b^	2.38b	2.28ab	2.27ab	3.80^bc^	3.77^b^	3.53^b^
B_1.5_	26.33^a^	25.31^ab^	23.98^b^	2.40ab	2.31b	2.29a	3.86^b^	3.80^b^	3.57^b^
B_2.0_	26.69^a^	26.02^a^	24.36^a^	2.43a	2.33a	2.31a	3.94^a^	3.85^a^	3.64^a^
Mean	25.24	24.66	23.09	2.38	2.29	2.27	3.83	3.76	3.532

	Reproductive stage in different crops*								
	**Cis**	**Fls**	**Frs**	**Fls**	**Cis**	**Frs**	**Fls**	**Cis**	**Frs**

B_0_	24.29^b^	24.14^b^	23.40^b^	2.34^c^	2.27b^c^	2.26^c^	3.78^bc^	3.74^c^	3.75^b^
B_0.5_	25.89^b^	25.16^ab^	24.30^b^	2.37^bc^	2.29b^c^	2.29^b^	3.80^bc^	3.78^bc^	3.78^b^
B_1.0_	26.74^ab^	26.04^ab^	24.98^ab^	2.39^bc^	2.30^b^	2.32^b^	3.83^b^	3.81^b^	3.82^ab^
B_1.5_	27.31^a^	26.54^ab^	25.55^a^	2.41^b^	2.33^a^	2.35^ab^	3.88^ab^	3.86^ab^	3.85^ab^
B_2.0_	27.79^a^	27.00^a^	25.89^a^	2.44^a^	2.34^a^	2.37^a^	3.94^a^	3.90^a^	3.91^a^
Mean	26.20	25.78	24.83	2.39	2.31	2.32	3.85	3.82	3.82

	**Maturity**								
B_0_	25.16^c^	24.72^bc^	24.09^c^	2.28^c^	2.25^c^	2.26^b^	3.74^b^	3.71^bc^	3.71^b^
B_0.5_	26.02^b^	25.72^b^	25.17^ab^	2.31^b^	2.28^ab^	2.27^b^	3.78^b^	3.74^b^	3.73^ab^
B_1.0_	26.69^b^	26.24^a^	25.80^ab^	2.33^b^	2.30^ab^	2.29^ab^	3.81^ab^	3.77^b^	3.75^ab^
B_1.5_	27.15^a^	26.61^a^	26.15^a^	2.35^b^	2.32^b^	2.32^a^	3.83^a^	3.81^a^	3.78^ab^
B_2.0_	27.29^a^	26.77^a^	26.72^a^	2.38^a^	2.35^a^	2.33^a^	3.84^a^	3.83^a^	3.82^a^
Mean	26.46	26.01	25.58	2.33	2.30	2.29	3.80	3.77	3.76

*Cis, curd initiation stage in cauliflower, Fls, flowering stage in cowpea; Frs, fruiting stage in okra. The means followed by a different letter are significantly different at *p* ≤ 0.05 by Duncan’s multiple range test (values are means of 3-replicates).

### Correlation between the soil properties and mean yield of cropping sequence

**FIGURE 3:**
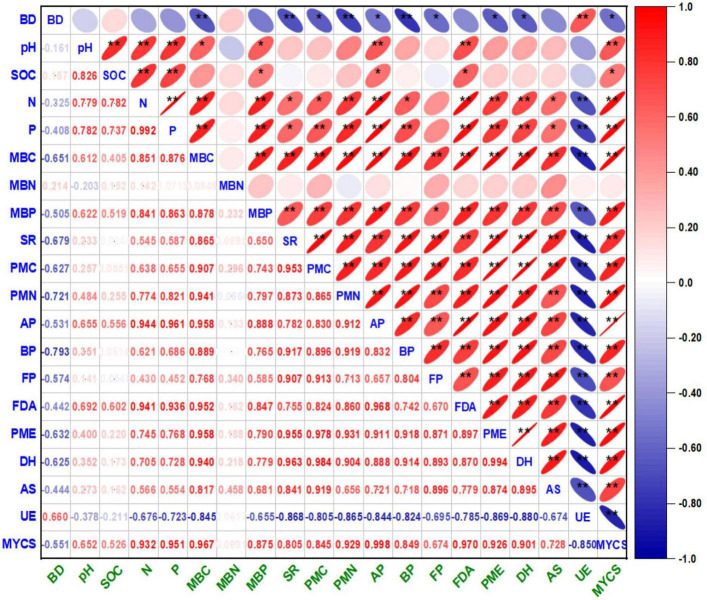
Correlograms of Pearson correlation coefficients (*r*) matrix between the soil physico-chemical and biological entities under cauliflower–cowpea–okra cropping system. The correlation coefficient (*r*) values are significantly positive at *p* < 0.01 (**) and *p* < 0.05 (*) levels of probability (2-tailed); the color assigned to a point in the correlograms grid indicates the strength of a correlation between the soil biological entities, and *r* values correspond directly to the color codes ranging from red to blue, respectively. Right and left tilted ellipse in the correlograms grid indicate positive and negative correlation, respectively. where, Bd, bulk density; SOC, soil organic carbon; MBC, microbial biomass carbon; MINN, mineralizable nitrogen; AP, actinomycetes population; BP, bacterial population; FP, fungal population; AS, arylsulphatase activity; DHA, dehydrogenase activity; FDA, fluorescein di-acetate hydrolysis activity; PMA, phosphomonoesterase activity; UE, urease activity.

The univariate correlation coefficients (r) in between the soil properties (physico-chemical and biological) and mean yield of cropping sequence (MYCS) are illustrated by the correlogram [Auto correlation function (ACF) plot] (Figure 3). In general, the results exhibited an existence of a significant positive correlation (*p* < 0.01 and *p* < 0.01) between the soil properties and MYCS barring MBN, at different crop growth stages, thereby signaling a synergistic relationship between them. However, BD and UE activity exceptionally showed a negative non significant correlation with the rest of the parameters, while both are positively and strongly correlated to each other (*p* < 0.01). Selectively, pH is highly correlated (*p* < 0.01) with SOC, available N and P, MBC, MBP, AP, FDA, and MYCS but positively correlated (*p* < 0.05) with PMN. Similarly, SOC showed a highly significant positive correlation (*p* < 0.01) with available N and P, FDA but exhibited a positive correlation (*p* < 0.05) with MBP, AP, and MYCS, respectively.

### Clustered analysis

**FIGURE 4:**
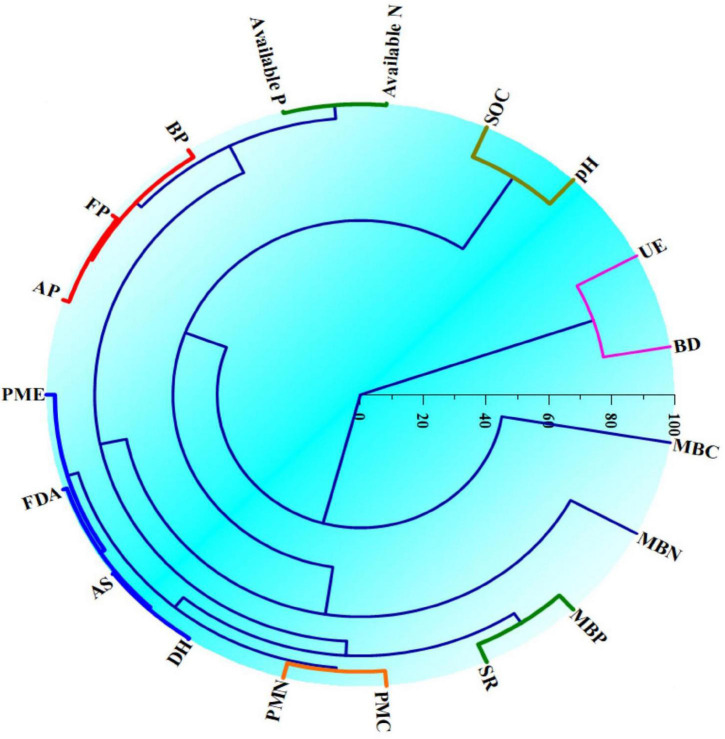
Hierarchical clustering of the soil biological entities indicating similarities in different soil physico-chemical and biological entities in cauliflower-cowpea–okra cropping system. MBC, microbial biomass carbon; SR, soil respiration; MINC, mineralizable carbon; MINN, mineralizable nitrogen; AP, actinomycetes population; BP, bacterial population; FP, fungal population; AS, arylsulphatase activity; DHA, dehydrogenase activity; FDA, fluorescein di-acetate hydrolysis activity; PMA, phosphomonoesterase activity; UE, urease activity; VS, vegetative stage; CIS, curd initiation stage; MS, maturity stages are the Blevels, respectively.

Hierarchical clustering (Figure 4) in respect of soil biological entities under different crops in CCOCS identified the distinct clusters based on similarity in function and other relevant biological attributes. In respect of cauliflower, three distinct clusters were formed *viz*., Cluster-I (PMC and PMN), Cluster-II (Microbiological pools of soil organic matter: MBN, MBC, and SR) and Cluster-III (Microbial population: AP, FP, and BP; Soil enzymes: AS, PMA, FDA, and DH). Similarly, in the case of cowpea, three distinct clusters were generated *viz*. Cluster-I (MBN, MBC, and SR), Cluster-II (PMC, PMN, and PMA), and Cluster-III (Microbial population: FP, AP, and BP; Soil enzymes: FDA, DH, and AS). Likewise in okra, similar clusters were formed *viz*. Cluster-I (PMC and PMN), Cluster-II (Microbiological pools of soil organic matter: MBN, MBC, and SR) and Cluster-III (Microbial population: FP, AP, and BP; Soil enzymes: FDA, DH, AS, and PMA). However, the UE enzyme formed a discrete outlier as this enzyme had a reciprocal relationship with the examined parameters (Figure 4).

### Principal component analysis

**FIGURE 5:**
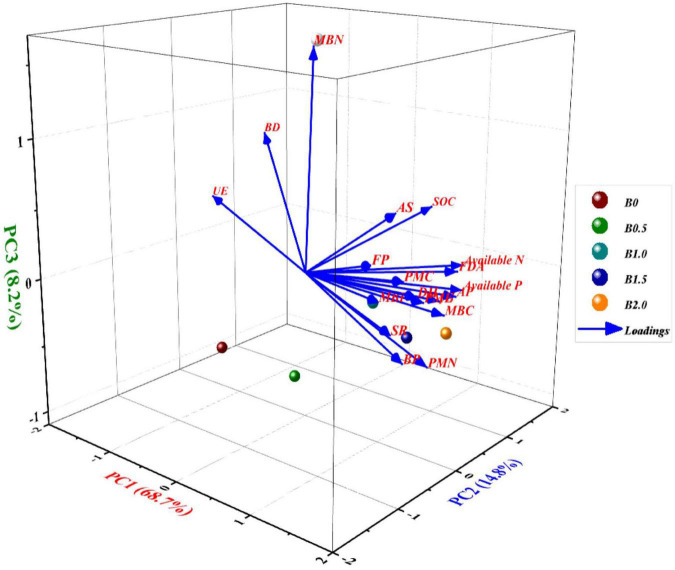
Three-dimensional graphical biplot showing the loading and score plot formed by principal components 1, 2, and 3 with different soil physicochemical and biological entities in cauliflower-cowpea–okra cropping system. Percentage values on PC1, PC2, and PC3 indicate the respective variance explained by the first three PCA axes; where, MBC, microbial biomass carbon; MINN, mineralizable nitrogen; AP, actinomycetes population; BP, bacterial population; FP, fungal population; AS, arylsulphatase activity; DHA, dehydrogenase activity; FDA, fluorescein di-acetate hydrolysis activity; PMA, phosphomonoesterase activity; UE, urease activity.

Principal component analysis executed in respect of soil physico-chemical and biological properties in CCOCS extracted three principal components with eigenvalues equal or greater than unity (Supplementary Table 3), accounting cumulatively up to 95.56% of the total variance since they possessed eigenvalues >1.0 and explained >5% of the variance in the total dataset of the available data (Supplementary Table 3). The loading plot (Figure 5), generated three PCs with eigenvalues equal or greater than unity *viz*. PC1 (68.7%), PC2 (14.8%), and PC3 (8.2%), respectively. Barring the soil physiochemical properties, the loading plot (Figure 5) (denoted by blue lines), elucidated that PC1 had large positive loadings on BP, MBC, MBN, MBP, PMC, and SR and subsequently followed by soil enzymes, and they were highly correlated to each other. Similarly, PC 2 exerted higher loadings on PMC and PMN, whereas PC 3 had heavy loadings on MBN and UE (Figure 5), respectively. Contrarily, UE activity was negatively correlated with PC1 which is attributed to the reciprocal relation with added corresponding B levels. In case of PC2, it showed heavy loading on PMA and PMC. The respective score plots (denoted by red colored dots) of the crops in CCOCS were divided into four quadrants (I, II, III, and IV) based on component (1 and 2) scores (Figure 5) to allow for better visual discrimination of B levels on soil physico-chemical and biological properties in CCOCS. The scoreplot showed that the first quadrant identifies that the B levels of 1 and 1.5 kg ha^–1^ displayed positive heavy loading on some PC1 components *viz*. soil chemical properties (SOC and pH), soil enzymes (FDA, PMA, DH, and AS) and microbial population (AP and FP), respectively. Similarly, the 2*^nd^* quadrant, indicated that B level of 0.5 kg ha^–1^ had greater loadings on BD and UE wherein both these variables had reciprocal relation with the rest of the studied parameters. The 3rd quadrant was occupied by control (B level of 0 kg ha^–1^) which did not influence any soil parameters. Interestingly, the 4*^th^* quadrant harbored the most important parameters influencing the crop yield in the sequence namely BP, MBC, MBN, MBP, PMN, PMC, SR, respectively in the biplot (Figure 5), which in turn, was affected by higher B level of 2 B kg ha^–1^.

### Stepwise multiple linear regressions for predicting the best model for crop yield

The stepwise multiple linear regressions (SMLR)exercised on MYCS showed the best fitting model that may produce maximum yield is enlisted in Table 7. The results of SMLR implied that the retained biological entities BP, MBC, MBN, MBP and PMC (Table 8) were the best predictors contributing 44.8, 22.17, 18.67, and 14.18%, respectively to the MYCS. Fitting MYCS as a dependent attribute (response variable) and biological properties as the independent attributes (predictor variables) (Eq. 1), a best-fitting regression model was generated below as:


(1) MYCS=11.97+1.625BP+0.760MBC+0.494MBN+0.0843MBP

**TABLE 7:** Model summary of SMLR under cauliflower-cowpea-okra cropping system.

S	R^2^	R^2^ (adjusted)	PRESS	R^2^ (predicted)	Residuals	Durbin-Watson statistic
0.32	99.74%	99.64%	2.08	99.47%	2.18	1.84

**TABLE 8:** Stepwise regression variances analysis of different soil biological properties in cauliflower-cowpea-okra cropping system.

Source	DF	Seq SS	Contri- bution	Adj SS	Adj MS	F-value	*P*-value	Coef	SE Coef
Regression	4	394.54	99.74%	394.54	98.63	966.30	0.00	11.97	3.18
BP	1	388.07	44.8%	4.05	4.05	39.71	0.00	1.63	0.26
MBC	1	4.74	22.17%	2.51	2.51	24.61	0.00	0.76	0.15
MBN	1	1.04	18.67%	1.49	1.49	14.62	0.00	3.49	0.91
MBP	1	0.69	14.18%	0.69	0.69	6.80	0.03	-0.08	0.03
Error	10	1.02	0.26%	1.02	0.10				
Total	14	395.55	100.00%						

Stepwise selection of terms: α to enter = 0.15, α to remove = 0.15. S, standard error of the regression; PRESS, predicted residual error sum of squares; Seq SS, sequential sums of squares; Adj SS, adjusted sum of squares; Adj MS, adjusted mean squares; SE Coef = Standard error of the coefficient.

### Path analysis

**FIGURE 6:**
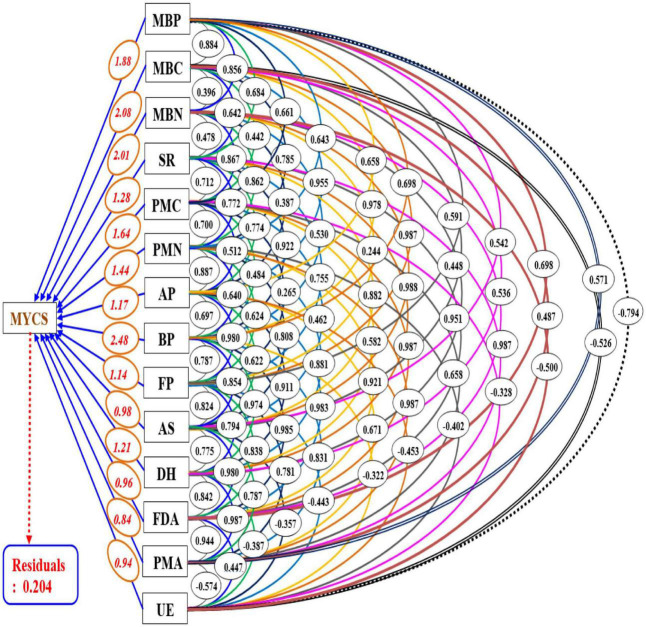
Path diagram depicting the contribution of soil biological entities to the MYCS in cauliflower-cowpea–okra cropping system. Single-headed arrows, double-headed arrows and connectors signify the path coefficient (β) (direct effect), simple correlation coefficients between variables and mutual association, respectively; where, MYCS, mean yield of the cropping system; MBC, microbial biomass carbon; MBN, microbial biomass nitrogen; SR, soil respiration; PMC, potentially mineralizable carbon; PMN, potentially mineralizable nitrogen; AP, total actinomycetes population; BP, total bacterial population; FP, total fungal population; AS, arylsulphatase activity; DH, dehydrogenase activity; FDA, fluorescein di-acetate hydrolysis; PMA, phosphomonoesterase activity; UE, urease activity.

For the execution of path analysis, all the biological entities were assigned as predictor variables excluding soil physiochemical properties, while the MYCS under CCOS was assigned as a response variable to avoid the redundancy of data. In path analysis, the magnitude of the contribution of all the biological entities to MYCS was quantified by its corresponding path coefficient values. Results of the path analysis (Figure 6) showed that BP with path coefficient values of 2.09 had the highest and most significant direct effect on MYCS and had twelve numbers of indirect effects emanating from the rest of the twelve biological parameters under investigation. Barring, urease enzymes, these twelve biological parameters indirectly contributed to MYCS by largely linking to the BP and their indirect path coefficients through BP. The contributions and impact of the biological entities to MYCS can be ranked in decreasing order as BP > MBC > MBN > MBP > PMC > PMN > SR > DH > AP > FP > AS > FDA > UE > PMA, respectively.

## Discussion

Boron is one of the indispensable nutrients for the ideal growth, development, produce, and quality of crops (Shireen et al., 2018). In general, B being a vital nutrient plays a role in plant growth, phenols, lignification, tissue expansion, membrane-related reactions, ribose nucleic acid (RNA) metabolism, hydrocarbon metabolism, pollen germination and seed development which are directly implicated in increasing crop yield (Goldbach and Monika, 2007). The enhancement in crop yield as a result of B-fertilization could be ascribed to the improved availability and accessibility of nutrients to plants (Kumar et al., 2016, 2017), hence producing and mobilizing surplus carbohydrates and proteins along with its role in enhancing their translocation from the site of synthesis to the storage organs (Takkar and Randhawa, 1978; Verma et al., 2012). Moreover, B acts as a key role in many metabolic processes such as cell wall differentiation, cell development, N-metabolism, fertilization, fat metabolism, hormone metabolism, active salt absorption, and photosynthesis (Nason and McElory, 1963), which in turn contributed to higher fresh and dry matter yield of cauliflower. Similar findings in okra were also reported earlier (Saha et al., 2010; Rahman et al., 2017). Kumar and Sen (2004) reported that the application of B and Zn improved the yield and quality of okra seed. The beneficial impacts of B on curd quality and yield of cauliflower were acknowledged by Gupta (1993).

The decrease in BD under CCOCS might be due to an improvement in soil structure and porosity due to the addition of FYM. A slight increment in soil pH could be possibly due to the ligand exchange between OH^–^groups of soil Al and Fe(OH)_2_ and organic compounds, and the disintegration and binding of organic complexes of the applied FYM (Xu et al., 2006). Similarly, SOC in soil remained significantly (*p* ≤ 0.05) (Table 1) unaffected after two years of B application, however, an increment in SOC was observed possibly due to the SOC build-up through regular addition of FYM and desirable changes in biochemical and physical properties of soil (Ghosh et al., 2012; Bhupenchandra et al., 2022; Harish et al., 2022; Kumar et al., 2022). Also, another reason for the improvement in SOC could be due to the formation of a strong diol complex of B with organic matter in soils and the capacity of organic matter to improve CEC of soils (Bhupenchandra et al., 2021b). Increase in available N could be due the release of mineralized N by the addition of organic matter along with the concurrent release of N via symbiotic biological N fixation by cowpea roots, since B plays a vital role in biological N fixation and upsurges the number of effective nodules (Bolaños et al., 2001). The improvement in the status of available P could be explicated by the existence of positive interaction between P and B in the soil as both are in anionic forms and might have been involved in anion exchange (Bhupenchandra et al., 2021b).

Microbial biomass carbon is the measure of the C present within the living constituent of soil organic matter. Soil respiration (SR) is the CO_2_ released by the biological activities of soil organisms, involving plant roots, microbes, and soil animals are usually calculated as a flux of CO_2_ from the soil surface. Escalating B appliance quickly altered soil MBC content and soil respiration (Bilen et al., 2011). Improvement in MBC and CO_2_-C production in soil with the application of graded B could also be due to the continuous application of FYM in all the crops for two years in the sequence, which in turn, might have created a conducive atmosphere for intensified microbial activities in soil (Singh et al., 2020, 2021). Soil MBC, being an active and labile component of SOC dependent on the SOM (Chen et al., 2005; Kumar et al., 2022). There is no direct evidence of the effect of B application on microbial properties of soil. But, the enhancement in these biological properties with the incremental doses of B indicated a possible relationship between the B-fertilization and microbial activity of the soils. Subsequently, microbes are implicated in the assimilation of SOM, which further led to the release of B from organic complexes in soil (Kumar et al., 2016, 2017). Also, total B present in the soil fraction is closely related to SOM and was unleashed via microorganism action (Berger and Pratt, 1963). Upon intensifying the B appliance, the soil microbial biomass load in the soil quickly transformed and escalated vibrantly. Moreover, the accessibility of readily mineralized C and N, and improvement in soil physico-chemical properties might have enhanced the microbial population in soil (Bhardwaj and Datt, 1995; Kumar et al., 2022). Application of FYM improves the SOC pool by supplying organic matter in greater mineralizable form, thereby, delivering substrate for microbial utilization and this could be the cause of higher MBC and SR in the current study (Goldberg, 1997; Liu, 2000; Rajpoot et al., 2021).

The PMC, also known as biodegradable C, is the entirety of organic matter which can be decomposed through microbial action (Guo et al., 2019). Potentially mineralizable-N (PMN) is a measure of the active fraction of soil organic-N, predominantly accountable for the discharge of mineral-N via microbial accomplishment (Campbell and Curtin, 2007). The PMN is availed to plants and microorganisms in the form of NO_3_^–^ by aerobic mineralization. It is a fraction of N linked to the microbial biomass which is positively related to MBC. With crop growth, PMN content in soil is augmented as it is the quantum of N that mineralize with time at the most favorable temperature and moisture. It comprises a diverse group of organic complexes which encompass microbial biomass, crop residues and humus. The increment of PMC content might be attributed to the soil application of well-decomposed FYM which acts as a substrate for microbial entities. The enhanced N-mineralization was observed during the symbiotic biological N-fixation by cowpea roots since B acts a vital role in the biological fixation of N and augments the number of effectual nodules (Bonilla et al., 1997; Bolaños et al., 2001), and hence, might have created signaling compound through the rhizobia infection on roots of legume crop (Dénarié and Cullimore, 1993; Spaink, 2000).

It is established that B is vital for symbiont/plant signaling, namely *nod*-gene activation by root plant exudates and nodule invasion (Redondo-Nieto et al., 2001). Moreover, B is essential for infectivity thread advancement and nodule initiation (Bolaños et al., 1996) due to the function of B as a modulator of the interactions amidst plants derived infection thread matrix glycoproteins and the bacteria cell surface. Boron stabilizes membranes, which aids the relationship between bacterial cell surfaces and the peri-bacteroid membranes, helping them in regulating symbiotic setup (Bolaños et al., 1996). Specifically, B is indispensable for the target of nodule-specific plant-derived glycoproteins that are critical for signaling bacteroid differentiation into a N_2_-fixing form (Bolaños et al., 2004b). Thus, all these factors created a conducive atmosphere for augmenting the microbial population in the soil. Several B tolerant bacterial strains belonging to the genus *Bacillus, Chimaereicella, Pseudomonas, Microbacterium, Shewanella, Mycobacterium, and Rhodococcus* have been reported with the ability to accumulate B from soil (Ahmed et al., 2007; Raja and Omine, 2013). There are reports on increased rhizosphere microbial populations by B in soybean (Sun et al., 2013). There were reports of B improving the population of diverse bacterial orders (Burkholderiales, Nitrosospherales, and Rhodospirales) (Vera et al., 2019). Boron aids in the enhancement of endomycorrhizae in roots owing to the action of indole 3-acetic acid (IAA) oxidase activity that activates IAA intensities eventually augmenting the translocation of carbohydrates to roots thereby improving fungi–mycorrhizal interaction and its fungal population (Lambert et al., 1980; Kumar et al., 2017). Related findings were reported by Bilen et al. (2011), where the highest population of bacteria and fungi production were observed with 2 kg B ha^–1^ in altered growth periods of the plant and diverse soil depths.

Even though the direct role of the effect of B on soil enzymes could not be established, however, the improvement in the status of enzyme activities (AS, DH, FDA, and PMA) was observed during the two years of experimentation. Possibly it was speculated that the enhanced enzyme activities could be attributed to improved soil condition due to the continuous addition of organic matter in the form of FYM in all the crops for two years which enhanced greater microbial activities. Since mineralised C and N from FYM enhanced the soil physico-chemical properties and the quantum of applied-B, the microbial populace and soil enzyme activities increased (Bhardwaj and Datt, 1995; Kumar et al., 2022). The improvement in the soil enzymatic activities might be attributed to the readily degradable organic matter added to the soil, which increases soil microorganisms and soil enzyme activities (Perucci, 1992). Also, DH enzyme activities in soil improved under graded B-fertilization. A similar finding was reported by Bilen et al. (2011) who observed a significant (*p* < 0.01) positive correlation with B and DH enzyme activity. DHA is a key indicator of microbial activity and organic matter stability since it is directly implicated in microbial respiration (Nikaeen et al., 2015). Improvement in the rhizosphere soil enzyme activity of soybean on the appliance of B was also reported (Goldberg, 1997; Liu, 2000). It was reported that surface residues encouraged the conservation of mineralizable C via residues that bettered the activities of soil enzymes ensuing in higher soil microbial biomass carbon and enhanced soil quality (Mohammadi et al., 2012). The increment in the soil microbial populations enhanced the rhizosphere metabolisms and bettered the soil enzyme activities on the appliance of B (Sun et al., 2013). Urease (urea amidohydrolase, EC 3.5.1.5) is N-related extracellular enzyme, the enzyme implicated in the degradation of urea cleaving urea to NH_3_ and CO_2_ (Kappaun et al., 2018). Evidently, UE enzyme activity de-escalation in all the crops at different growth stages was observed in B applied to soil as compared to control. The most likely reason may be due to the fact that B containing acids acts as a UE inhibitor in soil (Vera et al., 2019). It was reported that UE activity was inhibited and minimized by the application of higher B levels. Furthermore, this profound effect of B on the UE enzyme activity might be attributed to its action on water-soluble N, apart from the structural impairment to the enzyme and improved availability of N, as amino-boranes, which might have inhibited the urease activity via feedback mechanism (Kappaun et al., 2018). It was reported that the enzyme UE was immobilized on a membrane of microbes as inhibited B (Zaborska, 1995).

Soil functional diversity is governed by substrate amount, quality, and microbial accessibility. (Bending et al., 2002). Consequently, the plots which received higher C sources could alter microbial load of organic matter and the functional diversity of the microbial community in soils (Sall et al., 2006). Higher H values observed under the plots receiving escalated 2 kg B ha^–1^ might be due to the higher receipt of B dose which in turn improves SOC contents in the soil as there existed a positive correlation between B and SOC had been earlier documented (Bilen et al., 2011). Similarly, in the current investigation also, higher SYI values hold true in those plots where the highest B levels were applied and this is attributed to the greatest C availability by catabolic diversity (Lagomarsino et al., 2011) ensuing in better soil functional diversity.

The forming of clusters (Figure 4) with MBC, MBN, and SR in the soil in CCOCS may be possibly due to the fact that MBC is on the whole readily decomposable pool of organic material owing to the simplest structure and high quality of C and nutrients where soil bacteria used to nourish (Singh et al., 1992). The primary activation of microbial activity possibly ensued from swift catabolism of simple soluble C compounds, thereby, augmenting microbial biomass load in soil (Singh and Singh, 1991). Due to the possibility of similarity in their function, the soil enzymes were found to accumulate in similar clusters in the soil.

Normally, the first component explains most of the variability contained in the data set (Johnson and Wichern, 2002). The loading plot (Figure 5) exhibited that BP, MBN, PMC and MBC, PMN, actinomycetes, soil enzymes barring urease and subsequently followed by FP, and are highly correlated to each other in CCOCS. Because these parameters are highly correlated to each other as the angle within the variables of 0 or 180° reveals a correlation of 1 or −1, respectively (Kohler and Luniak, 2005). However, UE activity was negatively correlated with PC1 which is ascribed to the inverse link between levels (Vera et al., 2019). Results of the path analysis (Figure 6) also showed that barring urease enzymes, all twelve biological parameters indirectly contributed to MYCS by largely linking to the BP and their indirect path coefficients through BP. The contributions of the biological entities to mean yield of the cropping sequence followed the trend of BP > MBN > PMC > AP > PMC > PMN > AP > BP > FP > AS > DH > FDA > PMA > UE, respectively. The execution of SMLR regression models is considered best fitting (*R*^2^ > 0.9), as, R^2^ is in the range of 0.90 and 1 (Ogwueleka and Ogwueleka, 2010). In general, an *R*^2^ value ranging between 0.8 and 0.9 implies a good fitting and values lesser than 0.8 signify a poor model.

## Conclusion

The results of the foregoing study revealed a tangible and significant impact of the graded levels of B-fertilization on soil biological entities under cauliflower-cowpea-okra cropping system in an acid Inceptisol. The key biological properties like BP, MBC, MBP, PMC, microbial population, and soil respiration were enhanced significantly with the incremental dose of B-fertilization. The order of the B treatments in respect of MBC, MBN and soil respiration at different crop growth stages was 2.0 kg B ha^–1^> 1.5 B kg ha^–1^> 1.0 B kg ha^–1^> 0.5 B kg ha^–1^> 0 kg B ha^–1^, respectively. Higher recoveries of PMC and PMN were noticed under 2 kg B ha^–1^ in cauliflower, cowpea and okra, respectively, at all the crop growth stages over control. Barring urease, the activities of all other important soil enzymes (AS, DH, FDA, and PMA) were increased significantly up to the application of 2 kg B ha^–1^. The positive impact of B-fertilization on these biological properties was observed at different growth stages of all three crops in the sequence which ultimately led to higher and sustainable crop production. A significant and positive relationship between these properties and crop yield greatly supported this observation. Multivariate analysis also confirmed the role of B-fertilization in the augmentation of the soil’s biological properties and yield enhancement. Overall, it was concluded that different soil physico-chemical and biological properties under the cauliflower-cowpea-okra cropping sequence can be invariably improved by the application of graded levels of B up to 2 kg B ha^–1^ in an acid Inceptisol. Future research entails more advanced research between B with soil microbial pools, microbial populations and soil enzymes to explore the precise mechanism of their interaction in soil. Comprehension of the mechanisms underlying established functions of B may explicate the significance of B and, in the end, lead to an advanced perception of its biological function, which has vital pragmatic implications in agriculture. The continuity of the residual impact as well as the beneficial effect of B-fertilization in such cropping sequences is therefore an important subject for future empirical research to elucidate its sustainability.

## Data availability statement

The raw data supporting the conclusions of this article will be made available by the authors, without undue reservation.

## Author contributions

IB: conceptualization, methodology formulation and implementation, data analysis, project administration, resource, and review. AB and DS: analysis and graphical works, editing, and results validation. AKC: data curation, reviewing, and editing. AdK and SC: editing of original and revised versions of the manuscript. AS, ED, GG, and LO: data collection and processing and original draft preparation. SB and MDS: review of literature and results compilation. MS and BAG: review of literature and basic analysis. AmK and SHD: results compilation and draft preparation. BG and MH: graphs and maps preparation. YD, KS, and ST: review of literature, results compilation, and English language correction. MR: review and editing of the final draft of manuscript, validation of statistical analysis, and formatting the manuscript. All authors contributed to the article and approved the submitted version.
