# Supplementary material for: Elucidating the impact of boron fertilization on soil physico-chemical and biological entities under cauliflower-cowpea-okra cropping system in an Eastern Himalayan acidic Inceptisol
Source: Front Microbiol. 2022 Nov 7;13:996220. doi: 10.3389/fmicb.2022.996220 (PMC9676249; doi:10.3389/fmicb.2022.996220)
Supplement: Supplementary file 1 [file Table_1.docx]

Supplementary Material

**Supplementary Table S1. Details of fie** Related Article**ld experiment on cauliflower-cowpea-okra cropping system.**

| **Particulars** | **Test crops** | | | |
| --- | --- | --- | --- | --- |
|  | **Cauliflower** | **Cowpea** | | **Okra** |
| Module of crop test: | To assess the direct effect of B-fertilization | To assess the residual effect of B-fertilization | | |
| Variety: | Girija | KSP-170 | Green Gold | |
| Plot size: | 4 × 3 m | | | |
| Spacing: | 55 cm × 40 cm | 40 cm × 20 cm | 45 cm × 30 cm | |
| Treatments: | 0, 0.5, 1.0, 1.5, 2.0 kg B ha^-1^ | B application exempted | | |
| NPK dose: | 80 kg N:26 kg P:50 kg ha^-1^ | 15 kg N:15 kg P: 8 kg K ha^-1^ | 50 kg N: 50kg P:50 kg K ha^-1^ | |
| FYM: | 10 Mg ha^-1^ | | | |
| Crop stages for observation: | Vegetative, curd initiation, maturity | Vegetative, flowering, maturity | Vegetative, fruiting, maturity | |
| Growing period: | October-January | February-April | May-July | |

**Supplementary Table S2. Initial soil physico-chemical and biological parameters.**

| **Parameters** | **Initial values** | **Methodology and citation** |
| --- | --- | --- |
| Soil bulk density (Mg m^−3^) | 1.25 | Clod method (Blake and Hartge 1986) |
| Soil pH | 4.82 | Potentiometric method (Page et al.1982) |
| Soil organic carbon (%) | 0.80 | Wet oxidation method  (Walkey and Black 1934) |
| Available nitrogen (kg ha^-1^) | 264.70 | Alkaline potassium permanganate method, Subbiahh and Asija (1956) |
| Available phosphorus (kg ha^-1^) | 14.48 | Bray’s method, Jackson (1973) |
| Microbial biomass C (MBC) (µg g^-1^) | 169.5 | - Chloroform fumigation-incubation method   (Jenkinson and Powlson 1976) |
| Microbial biomass nitrogen (MBN) (µg g^-1^) | 38.4 |  |
| Microbial biomass phosphorus (MBN) (µg g^-1^) | 4.04 | Brookes et al. 1982 |
| Soil respiration (SR) (μg CO_2_ day^-1^ g^-1^ FW) | 5.45 | Carbon dioxide testing method  (Zheng et al. 2000) |
| Potentially mineralizable C (PMC) μg C g^–1^ | 138.5 | Incubation method  (Anderson 2015) |
| Potentially mineralizable N (PMN)  (μg NH_4_-N g^–1^ day^–1^ soil) | 27.0 | Long-term aerobic incubation method (Campbell et al. 1993) |
| Total actinomycetes population (cfu g^-1^) | 14.4 | Spread soil dilution plate method  (Aneja 2003) |
| Total bacterial population (cfu g^-1^) | 7.0 |  |
| Total fungal population (cfu g^-1^) | 13.9 |  |
| Arylsulphataseactivity (AS) (μg*p*-nitrophenol g^-1^ h^-1^) | 7.9 | Colorimetry method  (Tabatabai and Bremner 1970) |
| Dehydrogenase (DH) μg TPF g^-1^ 24 hour^-1^) | 114.2 | Reduction of Triphenyl tetrazolium chloride (TTC)  (Casida et al. 1964) |
| Fluorescein di-acetate (FDA) hydrolysis  (μg fluorescein g^-1^ h^-1^) | 4.8 | FDA hydrolysis method  (Adam and Duncan 2001) |
| Phosphomonoesterase (PMA) activity  (μg*p*-nitrophenol g^-1^ hour^-1^) | 36.8 | Colorimetry method  (Tabatabai and Bremner 1969) |
| Urease activity(µg NH_4_-N g^-1^ soil 2 h^-1^) | 21.8 | Colorimetry method (Dick 1994) |

**Supplementary Table S3.** **PCA analysis of different soil physico-chemical and biological entities in cauliflower-cowpea-okra cropping system.**

| **Principal component** | | **PC1** | **PC2** | | **PC3** | | | |
| --- | --- | --- | --- | --- | --- | --- | --- | --- |
| Initial eigen  values | Total | 13.06 | 2.82 | | | 1.56 | |  |
|  | Percentage of variance | 68.72 | 14.83 | | | 8.21 | |  |
|  | Cumulative percentage | 68.72 | 83.55 | | | 91.75 | |  |
| Rotation sums of squared loadings | Total | 10.62 | 5.19 | | | 1.62 | |  |
|  | Percentage of variance | 55.91 | 27.33 | | | 8.52 | |  |
|  | Cumulative percentage | 55.91 | 83.24 | | | 91.75 | |  |
| **Eigen vectors^b^** | |  | | **Factor loadings^a^** | | |  | |
|  |  | **PC1** | **PC2** | | **PC3** | | | |
| BD | | *-0.785* | *0.016* | | *0.413* | | | |
| pH | | *0.115* | *0.907* | | *-0.190* | | | |
| SOC | | ***0.942*** | *0.964* | | *0.160* | | | |
| Available N | | ***0.968*** | *0.871* | | *0.082* | | | |
| Available P | | ***0.974*** | *0.844* | | *-0.008* | | | |
| MBC | | **0.964** | 0.547 | | 0.018 | | | |
| MBN | | **0.957** | -0.004 | | **0.952** | | | |
| MBP | | **0.944** | 0.222 | | 0.147 | | | |
| SR | | 0.817 | 0.122 | | 0.034 | | | |
| PMC | | 0.832 | **0.910** | | 0.226 | | | |
| PMN | | 0.827 | **0.833** | | -0.176 | | | |
| AP | | 0.710 | 0.679 | | 0.048 | | | |
| BP | | **0.971** | 0.227 | | -0.109 | | | |
| FP | | 0.687 | 0.023 | | 0.297 | | | |
| FDA | | 0.547 | 0.712 | | 0.133 | | | |
| PME | | 0.544 | 0.357 | | 0.120 | | | |
| DH | | 0.807 | 0.302 | | 0.153 | | | |
| AS | | 0.632 | 0.216 | | 0.453 | | | |
| UE | | 0.604 | -0.339 | | **0.839** | | | |

**Extraction Method**: Principal Component Analysis, **Rotation Method:** Varimax with Kaiser Normalization.

**^a^**Rotation converged in 5 iterations and, **^b^**Boldfaced factor loadings are considered highly weighted.
